# Prevalence and associated factors of HIV testing among reproductive-age women in eastern Africa: multilevel analysis of demographic and health surveys

**DOI:** 10.1186/s12889-021-11292-9

**Published:** 2021-06-29

**Authors:** Misganaw Gebrie Worku, Getayeneh Antehunegn Tesema, Achamyeleh Birhanu Teshale

**Affiliations:** 1 Department of Human Anatomy, College of Medicine and Health Science, School of Medicine, University of Gondar, Gondar, Ethiopia; 2 Department of Epidemiology and Biostatistics, Institute of Public Health, College of Medicine and Health Sciences, University of Gondar, Gondar, Ethiopia

**Keywords:** Multi-level analysis, HIV/AIDS, HIV testing, Eastern Africa

## Abstract

**Background:**

Despite efforts made to reduce the spread of the human immune-deficiency virus (HIV), its testing coverage remains low in low and middle-income countries (LMIC). Besides, information on factors associated with HIV counseling and testing among reproductive-age women is not sufficiently available. Therefore, this study was aimed to determine the pooled prevalence and factors associated with HIV testing among reproductive-age women in eastern Africa.

**Methods:**

Secondary data analysis was conducted based on the Demographic and Health Surveys (DHS) data conducted in East African countries. We pooled the most recent DHS surveys done in 11 East African countries. A total weighted sample of 183,411 reproductive-age women was included for this study. Both bivariable and multivariable multilevel logistic regression models were fitted. Variables with a *p*-value ≤0.2 in the bivariable analysis were selected for multivariable analysis. Finally, in the multivariable analysis, variables with a *p*-value ≤0.05 were considered as significant factors affecting HIV testing.

**Results:**

The pooled prevalence of HIV testing in eastern Africa was 66.92% (95%CI: 66.70, 67.13%). In the multivariable multilevel analysis factors such as the age of respondent, marital status, educational level, HIV knowledge, HIV stigma indicator, risky sexual behavior and women who visit a health facility were positively associated with HIV testing coverage among reproductive-age women. While women from rich and richest households, having multiple sexual partners, being from rural dwellers, late initiation of sex and higher community illiteracy level had a lower chance of being tested for HIV.

**Conclusion:**

The pooled prevalence of HIV testing in eastern Africa was higher than most previous studies. Age of respondent, residence, wealth index, marital status, educational level, HIV knowledge, stigma indicator, risky sexual behavior, women who visit a health facility, multiple sexual partnerships, early initiation of sex and community illiteracy level were significantly associated with HIV testing. There should be an integrated strategic plan to give education about methods of HIV transmission and the implication of HIV testing and counseling. So all the stakeholders should have an integrated approach by giving special attention to the factors that hinder HIV testing to increase awareness regarding the benefit of HIV testing and counseling to control the spread of HIV/AIDS.

## Background

Human immune deficiency virus (HIV) testing and counseling is a public health program concerned with diagnosing and minimizing the transmission of HIV/AIDS [1]. Currently, about 38 million people are living with HIV, nearly 36.2 million of them are adults and 1.8 million children [2]. Globally, 81% of all people living with HIV know their status and about 19 million remained unaware of their HIV status, which reduced to 7.1 million in 2019 [1]. The number of people who are newly infected with HIV declined from 3.4 million in 2011 to 2.1 million in 2013 [1]. Despite this progress, HIV/AIDS remains a major public health problem [3]. African countries, particularly eastern and southern regions are greatly affected by HIV/AIDS, which accounts for two-thirds of total new infections [4, 5].

Voluntary counseling and testing are considered as an initial step to detect, treat and prevent HIV/AIDS [6, 7]. The World Health Organization recommends HIV testing and counseling for all patients showing signs and symptoms of the disease [1, 8]. In Africa HIV testing coverage ranges from 33.5 to 82.3% [9–12].

According to studies conducted in different parts of the world age of the respondent, having multiple sexual partnerships, early initiation of sex, level of education, marital status and socio-economic status are some of the factors that are significantly associated with HIV testing [7, 10, 13, 14]. Besides, stigmatized attitudes, levels of knowledge about HIV/AIDS and risky sexual behavior have an association with HIV testing and counseling [7, 12, 13, 15–19].

Even though HIV testing is very crucial for all strategies related to care, prevention and treatment of HIV/AIDS, it is less practiced among reproductive-age women, particularly in developing countries [6, 16]. Every individual needs to know their HIV status for the benefit of themselves and others [1, 20, 21]. Although global efforts had made to reduce the spread of HIV/AIDS, its testing coverage remains low in developing countries [2]. The assessment and identification of factors that affect the utilization of HIV counseling and testing help policymakers to design effective strategies towards preventing and controlling HIV/ADIS. Besides, previous studies were conducted at a country level, most of them did not assess the community-level factors, which might be related to HIV testing coverage. Therefore, this study aimed to assess the pooled prevalence and factors associated with HIV testing among reproductive-age women in eastern Africa.

## Methods

### Data sources

This study was a secondary data analysis based on datasets from the most recent Demographic and Health Surveys (DHS) conducted in east Africa (Burundi, Ethiopia, Comoros, Uganda, Rwanda, Mozambique, Madagascar, Zimbabwe, Kenya, Zambia, and Malawi). The DHS is a nationally representative survey that collects data on basic health indicators like mortality, morbidity, family planning service utilization, fertility, maternal and child health. Each survey used a two-stage stratified sampling technique to select the study participants. We pooled the most recent DHS data done in 11 East African countries and a total weighted sample of 183,411 reproductive-age women was included for this study. The survey year and total weighted sample included for this study were presented in Table 1.

**Table 1:** Showing the survey year and total weighted sample for each country

Country	Year of survey	Weighted sample
Burundi	2016	17269
Ethiopia	2016	15683
Kenya	2014	31079
Comoros	2012	5329
Madagascar	2008	17375
Malawi	2015/16	24562
Mozambique	2011	3061
Rwanda	2352	13497
Uganda	2016	4264
Zambia	2018	16411
Zimbabwe	2013/2014	9955
Total		183,411

### Variables

The outcome variable of this study was “ever been tested for HIV” which was coded as “0” for no and “1” for yes.

The independent variables included in this study were categorized as individual and community-level factors. The individual-level factors included were: age of respondent, marital status, age at 1st sex, stigmatized attitude, educational level, household wealth status, HIV knowledge, risky sexual behavior, visiting of health facility and multiple sexual partnerships. The community-level factors were community illiteracy level and residence. The community illiteracy level of women was generated by aggregating the individual level factor women’s educational level by considering the proportion of women in the community that did not have formal education and by categorized this proportion as high and low based on the national median value.

### Operational definition

#### HIV knowledge

It was generated based on three questions related to HIV prevention and three questions related to the modes of HIV transmission and graded as low (if a woman answered ≤3 questions correctly), high (if a woman answered 4–5 questions correctly) or comprehensive (if a woman answered all the 6 questions correctly) [22].

#### Risky sexual behaviors

Assessed based on the five questions; having had STI in last 12 months, genital sore/ulcer in last 12 months, genital discharge in last 12 months, having at least one sexual partner other than the husband in the last 12 months, and multiple lifetimes sexual partnership. These were combined into an index of risky sexual behavior with three categories: “No risk” (if the response is no for all questions), “Some risk” (if the response is yes for one of the five questions) and “High risk” (if the response is yes to at least two questions) [22].

#### HIV stigma indicator

Six questions, which indicate negative attitudes towards people living with HIV / AIDS were used to this variable. This was categorized as “No stigma” (if we got a score of 6), “Low stigma” (if we got a score of 4–5), “Moderate stigma” (if we got a score of 2–3), and “High stigma” (if we got only score 1) [22].

### Data management and analysis

Data extraction, recoding and analysis were done using STATA version 14 software. Both descriptive and analytical analyses were conducted. The data were weighted before doing statistical analysis to restore the representativeness of the data and to get a reliable estimate and standard error. Because of the hierarchical structure of the DHS data, a multilevel binary logistic regression analysis was used. The Interclass Correlation Coefficient (ICC), Proportional Change in Variance (PCV) and Median Odds Ratio (MOR) were calculated to assess whether there was clustering or not. In this study, four models have fitted; the null model- a model without explanatory variables, model I- a model with individual-level factors, model II- a model with community-level factors and model III- a model with both individual and community-level factors. Model comparison was done based on deviance. Both bivariable and multivariable multi-level logistic regression were done. At the bivariable analysis variables with a *p*-value ≤0.2 were considered for multivariable analysis. Finally, variables with a *P*-value of ≤0.05 in the multivariable analysis were considered as a significant factor associated with HIV testing among reproductive-age women.

## Results

### Socio-demographic characteristics of study participants

A total weighted sample of 183, 411 reproductive-age women were included. About 21.54% of women were in the age group of 19 years and below. The majority (92.13%) of women initiated sex at an earlier age and more than half (56.82%) of them had visited health facilities in the last 12 months. Nearly, half (48.65%) of the study participants attained primary education. Regarding HIV knowledge, about 47.81% of respondents had comprehensive knowledge about HIV/AIDS and most (60.49%) of them had multiple sexual partnerships. Concerning risky sexual behavior, the majority (81.70%) of the participant had no risky sexual behavior and 71.96% of the participants were rural dwellers (Table 2).

**Table 2:** Sociodemographic characteristics of the respondents in eastern Africa (*N* = 183, 411)

Variables	Frequency (%)
Age (years)	
15–19	39,510 (21.54%)
20–24	34,150 (18.62%)
25–29	31,766 (17.32%)
30–34	26,992 (14.72%)
35–39	21,886 (11.93%)
40–44	16,406 (8.94%)
45–49	12,701 (6.92%)
Highest education level	
No education	33,035 (18.01%)
Primary education	89,229 (48.65%)
Secondary education	51,294 (27.97%)
Higher education	9,840 (5.37%)
Wealth index	
Poorest	32,495 (17.72%)
Poorer	33,755 (18.40%)
Middle	34,934 (19.05%)
Richer	37,225 (20.30%)
Richest	45,001 (24.54%)
Risky sexual behavior	
No risk	129,912 (81.70%)
Some risk	20,169 (12.68%)
High risk	8,931 (5.62%)
HIV knowledge	
Low knowledge	17,049 (10.03%)
High knowledge	71,694 (42.16%)
Comprehensive knowledge	81,301 (47.81%)
Marital status	
Unmaried	88,582 (48.30%)
Maried	94,829 (51.70%)
Number of sexual partners	
One	72,463 (39.51%)
More than one	110,948 (60.49%)
Stigma indicator	
No stigma	5,961 (7.15%)
Low stigma	35,612 (42.71%)
Moderate stigma	34,231 (41.06%)
High stigma	7,569 (9.08%)
Residence	
Urban	51,426 (28.04%)
Rural	131,985 (71.96%)
Age at sex	
Before 20 years	108,533 (59.17%)
At 20 and after years	74,878 (40.83%)
Visit health facility	
No	72,075 (43.18%)
Yes	94,857 (56.82%)
Community illiteracy level	
High	90,641 (49.42%)
Low	92,770 (50.58%)

### Prevalence of HIV testing in eastern Africa

**Fig. 1:**
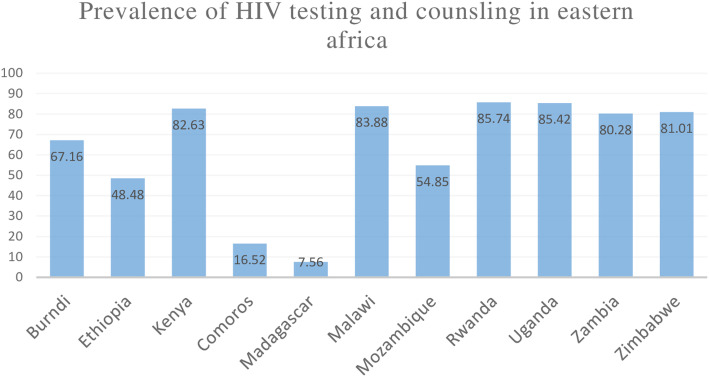
Showing the prevalence of HIV testing across different countries

The pooled prevalence of HIV testing in eastern Africa was 66.92% (95%CI: 66.70, 67.13%) ranged from 7.56% in Madagascar to 85.74% in Rwanda (Fig. 1).

### Random effect model and model fitness

**Fig. 2:**
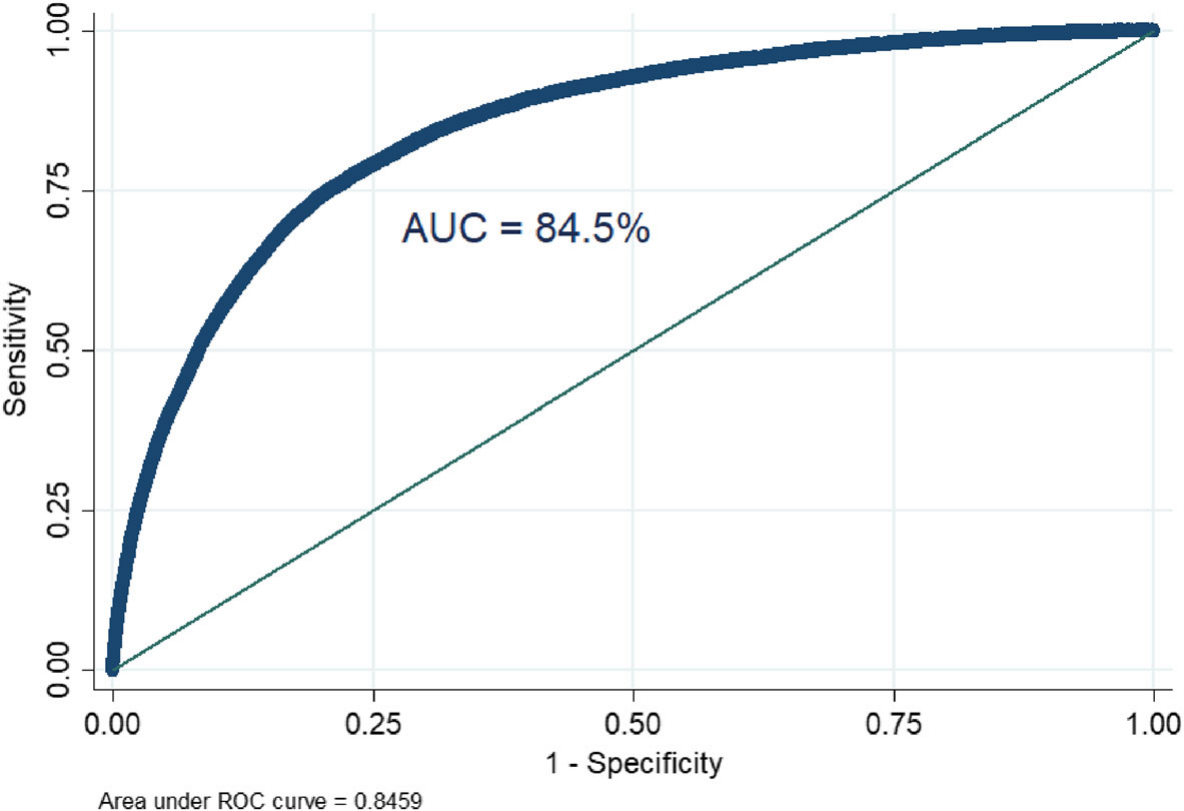
Showing the area under the ROC curve

As shown in Table 2, the ICC value in the null model was 0.10, which indicates that about 10% of the total variation in HIV testing coverage was attributable to cluster variability. Besides, the MOR value in the null model was 1.77, which indicates that there was a significant clustering of HIV testing among reproductive-age women. Furthermore, the PCV (0.36) in the final model (model III) indicated that about 36% of the variation in HIV testing was explained by both individual and community-level factors. The final model (Model III) was the best-fitted model since it had the lowest deviance (Table 3). The model predictive ability was assessed using the Area Under the Curve (AUC) and Receiver Operating Curve (ROC). These are plotted based on the probability of sensitivity and 1 – specificity. Accordingly, the AUC of the final model was 84.5% and indicated that the model’s ability to predict HIV testing was good (Fig. 2).

**Table 3:** Random effect model and model fitness for the assessment of HIV testing among reproductive-age women in eastern Africa

Parameter	Null model	Model I	Model II	Model III
Intraclass correlation coefficient (ICC)	0.10031	0.0692	0.0782476	0.066394
Percentage change in variation (PCV)	Ref	0.33	0.39	0.36
Median odds ratio (MOR)	1.77	1.6	1.65	1.58
Model comparison				
Log likelihood	− 113956.33	−75036.661	−113215.37	−74814.426
Deviance	227912.66	150073.322	226420.47	149628.852

### Factors associated with HIV testing

For assessing factors associated with HIV testing, we consider the final model since it had the lowest deviance. In the multivariable multilevel analysis factors such as the age of respondent, marital status, educational level, HIV knowledge, HIV stigma indicator, risky sexual behavior and women who visit a health facility were positively associated with HIV testing coverage among reproductive-age women. While women from rich and richest households, having multiple sexual partners, being from rural dwellers, late initiation of sex and higher community illiteracy level had a lower chance of being tested for HIV. Women aged 20 years and above had higher odds of being tested for HIV compared with women of 15–19 years old. The odds of testing for HIV was 2.67 (AOR = 2.67: 95%CI; 2.60, 2.74) times higher for women visiting health centers in the last 12 months compared with their counterparts. Regarding educational level, the odds of testing for HIV was 2.30 (AOR = 2.30: 95%CI; 2.22, 2.38), 2.39 (AOR = 2.39: 95%CI; 2.29, 2.50) and 3.13 (AOR = 3.13: 95%CI; 2.89, 3.39) times higher for women with primary, secondary and higher educational level, respectively, compared to women with no formal education. Being from rich (AOR = 0.93: 95%CI; 0.89, 0.97) and richest (AOR = 0.80: 95%CI; 0.76, 0.84) households had lower odds of being tested for HIV. Being married had 1.32 (AOR = 1.32: 95%CI; 1.28, 1.36) times more likely to be tested for HIV compared with their counterpart. Individuals who initiate sex after 20 years of age had 33% (AOR = 0.67: 95%CI; 0.65, 0.69) lower odds of being tested for HIV compared to those who initiate sex early. Regarding stigma, women with higher, moderate and low stigma scores had 1.56 (AOR = 1.56: 95%CI; 1.46, 1.66), 2.24 (AOR = 2.24: 95%CI; 2.16, 2.32), and 2.27 (AOR = 2.27: 95%CI; 2.19, 2.35) times higher odds of being tested for HIV compared to those with no stigma. Women with higher (AOR = 6.44: 95%CI: 6.21, 6.68) and comprehensive knowledge (AOR = 10.7: 95%CI; 10.29, 11.12) about HIV/AIDS had higher odds of being tested for HIV compared with women with low knowledge. Women having high (AOR = 1.78: 95%CI; 1.67, 1.90) and some risky sexual behavior (AOR = 1.59: 955CI; 1.53, 1.66) had higher odds of being tested for HIV compared to women with no risky sexual behavior. Women with multiple sexual partners had a 36% lower chance of being tested for HIV (AOR = 0.64: 95%CI; 0.62, 0.66) as compared to their counterparts. Women from the rural areas had 31% (AOR = 0.69: 95%CI; 0.67, 0.72) lower odds of being tested for HIV compared with their counterparts. Being from communities with higher community illiteracy levels had 27% (AOR = 0.73: 95%CI; 0.68, 0.78) lower odds of being tested for HIV compared with women from communities with lower community illiteracy levels (Table 4).

**Table 4:** The bivariable and multivariable multilevel binary logistic regression analysis of factors associated with HIV testing in East Africa in the final model

Variables	Ever tested for HIV		COR(95%CI)	AOR(95%CI)
	Yes	No		
Respondent age				
15–19	15383	24122	1	1
20–24	25379	8738	4.70 (4.55, 4.86)	3.67 (3.53, 3.82)*
25–29	25, 043	6664	6.33 (6.11, 6.55)	5.36 (5.12, 5.61)*
30–34	21074	5882	6.23 (6.01, 6.47)	5.49 (5.23, 5.76)*
35–39	16333	5521	5.20 (5.00, 5.40)	4.77 (4.54, 5.01)*
40–44	11673	4714	4.16 (3.99, 4.33)	4.12 (3.91, 4.34)*
45–49	7860	4827	2.78 (2.66, 2.90)	3.03 (2.87, 3.20)*
Visiting health facility				
No	34413	37624	1	1
Yes	74386	20401	4.06 (3.97, 4.15)	2.67 (2.60, 2.74)*
Highest educational level				
No education	17747	15253	1	1
Primary education	61029	28085	1.84 (1.79, 1.89)	2.30 (2.22, 2.38)*
Secondary education	35471	15781	1.81 (1.76, 1.87)	2.39 (2.29, 2.50)*
Higher education	8488	1347	4.58 (4.31, 4.87)	3.13 (2.89, 3.39)*
Wealth status				
Poorest	20082	12353	1	1
Poorer	21473	12245	1.25 (1.21, 1.29)	0.97 (0.93 1.02)
Middle	22840	12054	1.38 (1.33, 1.43)	0.98 (0.93 1.02)
Rich	25606	11585	1.56 (1.51, 1.61)	0. 93 (0.89, 0.97)*
Richest	32745	12231	1.71 (1.66, 1.76)	0.80 (0.76, 0.84)*
Marital statues				
Married	69948	24745	1.99 (1.95, 2.03)	1.32 (1.28, 1.36)*
Unmarried	52799	35722	1	1
No of sexual partner				
One	52493	19907	1	1
More than one	70254	40560	0.55 (0.54, 0.56)	0.64 (0.62 0.66)*
HIV knowledge				
Low knowledge	7414	9619	1	1
Higher knowledge	49482	22146	6.9 (6.69, 7.11)	6.44 (6.21, 6.68)*
Comprehensive knowledge	65773	15416	12.6 (12.20, 13.01)	10.7 (10.29 11.12)*
Stigma indicator				
Higher stigma	4375	3194	1.05 (1.01, 1.11)	1.56 (1.46, 1.66)*
Moderate stigma	25817	8414	2.30 (2.24, 2.37)	2.24 (2.16, 2.32)*
Low stigma	27813	7799	2.68 (2.61, 2.76)	2.27 (2.19, 2.35)*
No stigma	4905	1056	1	1
Residence				
Urban	38964	12404	1	1
Rural	83783	48063	0.66 (0.64, 0.67)	0.69 (0.67, 0.72)*
Risky sexual behavior				
Higher risk	7119	1805	2.21 (2.10, 2.33)	1.78 (1.67, 1.90)*
Some risk	15518	4636	1.80 (1.74, 1.87)	1.59 (1.53, 1.66)*
No risk	86043	43784	1	1
Age at sex				
Before 20 years	79591	29772	1	1
At 20 and above years	35500	9309	8.66 (8.37, 8.96)	0.67 (0.65, 0.69)*
Community illiteracy level				
Low	64381	26126	1	1
High	58366	34341	0.52 (0.48, 0.55)	0.73 (0.68, 0.78)*

*p ≤ 0.05

## Discussion

The pooled prevalence of HIV testing in east Africa was 66.9% (95%CI; 66.70, 67.13%), which varies greatly from region to region. The regional variations in quality and access to HIV testing facilities as well as knowledge related to HIV / AIDS may be the reasons for the reported regional variations of HIV testing in eastern Africa [9, 14]. The prevalence reported in this study was in line with the report in Kenya [12]. The finding in this study was greater than the study conducted elsewhere [3, 16, 23] and it was smaller than reports from different studies [9, 10]. The observed variations in the prevalence of HIV testing might be explained by cultural beliefs and lifestyle differences across regions [14]. Besides, the discrepancy might be due to the difference in quality and accessibility of HIV testing facilities [9, 14, 24, 25].

In this study age of respondent, multiple sexual partnerships, marital status, visiting health facility, stigmatized attitude towards HIV/AIDS, HIV knowledge, risky sexual behavior, residence, educational level, wealth status, age at first sex, and community illiteracy level were significant factors associated with HIV testing.

The odds of HIV testing was higher among married women compared with their counterpart. This finding was in agreement with the study done in Ethiopia [22]. This might be due to compulsory counseling and testing promotion for couples intending to get married by different organizations including religious groups [22]. Women who visit health care facilities had higher odds of being tested for HIV, which was supported by another study [16]. This might be because health professionals initiate people who visited health facilities for HIV testing [16].

In this study women from higher socioeconomic status had lower odds of being tested for HIV, which is contrary to another study [7]. This is justified by being rich may be associated with a greater awareness of risks and with reduced financial barriers to testing [26]. Women with primary and above educational levels had a higher chance of being tested for HIV, which is supported by different studies [16, 27]. The reason for this discrepancy might be as education can improve HIV knowledge as well as empowers women to make decisions to visit the health facility and use health services [28].

Women who had multiple sexual partners had less chance of being tested for HIV compared with their counterparts. However, the finding of this study was in contrast to another study [16]. This difference might be associated with individuals with a history of multiple sexual partnerships who might be fear of having HIV and have no interest to know their status. The study at hand revealed that women with higher and comprehensive knowledge about HIV had higher odds of being tested for HIV. This is supported by a study conducted in South Africa [13]. Different studies reported people with higher HIV-associated stigma scores had less chance of being tested for HIV. This is explained as people could be hesitant to test because the disclosure of a positive HIV test result may lead to loss of friendship, family relations, jobs and housing and health care due to stigmatization [2, 18, 22]. However, in the present study people with stigmatized attitudes had a higher chance of being tested for HIV and this might be explained by the variation in the cultural and socioeconomic status of the population included in this study.

In this study, women with risky sexual behavior had higher odds of being tested for HIV. This is supported by another study [22]. Individuals with risky sexual behavior live under persistent fear and uncertainty about their serostatus and are usually suspicious and worried that they might have infected with HIV. This urges them to develop habits of seeking VCT service [17].

Individuals who initiated sex early had a higher chance of being tested for HIV and this is supported by the study conducted in Malawi [14]. This might be explained as early age at first sexual intercourse is associated with a higher risk of acquiring the different sexually transmitted disease and risky sexual behaviors that may lead to a higher risk for HIV infection, which in turn enforce them to know their HIV status [29].

Women from rural dwellers had lower odds of being tested for HIV, which was supported by the study conducted in Ethiopia [16]. This may be justified by the better availability and accessibility of HIV testing facilities in urban settings compared with rural [30, 31]. Women from communities with higher illiteracy levels had less chance of being tested for HIV. This finding was supported by studies conducted in Ethiopia and Zambia [10, 16]. This might be associated with educational attainment may increase uptake of testing through increased recognition of the importance of knowing one’s HIV status [20, 32].

### Strength and limitation of the study

The study was based on weighted nationally representative data from 11 eastern African countries with a large sample size. Also, the analysis used the multilevel analysis to accommodate the hierarchical nature of the DHS data. Moreover, since it is based on the national survey data, the study has the potential to give insight for policy-makers and program planners to design appropriate intervention strategies both at the national and regional levels. However, this study had limitations in that the DHS survey was based on respondents’ self-report, this might have the possibility of recall bias. Besides, since this study was based on cross-sectional collected DHS data, we are unable to show the temporal relationship between HIV testing and independent variables.

## Conclusion

The prevalence of HIV testing in this study was higher than the report from previous studies. Age of respondent, marital status, educational level, HIV knowledge, stigma indicator, risky sexual behavior, women who visit a health facility, early initiation of sex, wealth status, multiple sexual partnerships, residence and community illiteracy level were significantly associated with HIV testing among reproductive-age women. Therefore, there should be an integrated strategic plan to give education about methods of HIV transmission and the implication of HIV testing and counseling.

Also, it is instrumental to bringing behavioral change, reducing unprotected sex and helping to reduce early initiation of sex and avoidance of having multiple sexual partners. So all the stakeholders should have an integrated approach by giving special attention to the factors that hinder HIV testing to increase awareness regarding the benefit of HIV testing and counseling to control the spread of HIV/AIDS.

## Data Availability

Data is available online and you can access it from www.measuredhs.com.

## Abbreviations

CI: Confidence Interval
CSA: Central Statistical Agency
DHS: Demographic Health Survey
EA: Enumeration Area
ICC: Intraclass correlation coefficient
LLR: likelihood Ratio
WHO: World Health Organization
